# Associations between family social circumstances and psychological distress among the university students of Bangladesh: To what extent do the lifestyle factors mediate?

**DOI:** 10.1186/s40359-021-00587-6

**Published:** 2021-05-16

**Authors:** Md. Nazmul Huda, Masum Billah, Sonia Sharmin, A. S. M. Amanullah, Muhammad Zakir Hossin

**Affiliations:** 1 School of Population Health, The University of New South Wales, Sydney, Australia; 2 School of Liberal Arts and Social Sciences, Independent University, Dhaka, Bangladesh; 3 Department of Sociology, East West University, Dhaka, Bangladesh; 4 Research and Evaluation, Take Two, Berry Street, Victoria, Australia; 5 Department of Occupational Therapy and Social Work and Social Policy, La Trobe University, Melbourne, Australia; 6 Department of Sociology, University of Dhaka, Dhaka, Bangladesh; 7 Department of Global Public Health, Karolinska Institute, Tomtebodavägen 18, 17177 Stockholm, Sweden; 8 Department of General Education, Eastern University, Dhaka, Bangladesh

**Keywords:** Mental health, Socio-economic position, Lifestyle, Mediation, Students, Bangladesh

## Abstract

**Background:**

While there is a growing body of empirical studies focusing on the social and behavioral predictors of psychological health, the mechanisms that may underlie the reported associations have not been adequately explored. This study aimed to examine the association of social and lifestyle factors with psychological distress, and the potential mediating role of the lifestyle factors in the estimated associations between social circumstances and psychological distress.

**Methods:**

A total of 742 tertiary level students (53% females) from a range of socio-economic backgrounds and multiple educational institutions participated in this cross-sectional study. The 12-items General Health Questionnaire (GHQ-12) was utilized for measuring psychological distress. Data related to students’ socio-demographic characteristics, family social circumstances, and lifestyle factors were also collected. Modified Poisson regression analysis was used to estimate the risk ratios (RR) and their 95% confidence intervals (CI).

**Results:**

The multivariable regression analysis suggests heightened risks of psychological distress associated with low parental Socio-Economic Position (SEP) (RR: 1.36; 95% CI: 1.07, 1.76), childhood poverty (RR: 1.31; 95% CI: 1.11, 1.55), and living away from the family (RR: 1.28; 95% CI: 1.07, 1.54). Among the lifestyle factors, past smoking, physical inactivity, inadequate fruit intake, and poor sleep quality were strongly associated with psychological distress and these associations persisted when the family social circumstances and lifestyle factors were mutually adjusted for. The lifestyle factors did not considerably mediate the estimated associations between family social circumstances and psychological distress.

**Conclusion:**

The social and lifestyle factors operated independently to increase students’ risk of psychological distress. Accordingly, while promoting students’ healthy lifestyles may reduce the overall burden of psychological distress, any equity initiative aiming to minimize the social inequalities in psychological health should be targeted to improving the living conditions in early life.

## Background

Psychological distress, especially among the students, has been a major public health concern because it is often associated with reduced academic performance [1], worse physical health [2], increased risk of mortality [3], and elevated healthcare costs [4]. Existing evidence indicates high prevalence rates of psychological distress among students in low, middle, and high-income countries. A nationwide Finnish study reported an increasing trend of psychological distress in students, from 22% in 2000 to 26% in 2004 to 28% in 2012 [5]. Upward trends in psychological distress were also observed among Norwegian students [6]. Similarly, a review of available studies in Bangladesh [7], Ethiopia [8], and Pakistan [9] suggests increasing rates of psychological distress in students, ranging from 41% to 73%. The growing rates indicate that psychological distress in students is a global concern.

There is plenty of evidence of links between socio-economic position (SEP) indicators (income, education, occupation, poverty, and living conditions) and psychological distress in students worldwide. For example, a systematic review reported associations between psychological distress and a range of social and economic risk factors including limited family income [10]. In addition, students who had low socio-economic conditions—having poor economic situation, living in rental apartments, and having less educated parents—reported higher levels of mental health problems such as depression, anxiety, and stress [11]. Older students, female students, students from rural areas, and students with low family income were also more likely to report psychological distress [12]. In addition, the socio-demographic profile of the parents—including low education, divorced relationship status, and father’s unemployment status—was related to students’ psychological distress [13]. Conversely, some studies found null associations between psychological distress and students’ socio-demographic characteristics [14] and parents’ socio-economic profile [15]. Thus, the existing body of published studies suggest that the associations of psychological distress with the socio-economic characteristics of students and their parents differ across studies, ranging from very strong to null associations.

Numerous studies also demonstrated associations between students’ psychological distress and lifestyle factors, including substance use, dietary patterns, smoking, sleep quality, and physical activity. For instance, studies conducted among students indicated that the chance of experiencing psychological distress significantly decreased with adequate sleep [16, 17] and higher fruit consumption [18]. A multi-country study showed that the risk of psychological distress was significantly greater among adolescent students who drank alcohol than those who did not [19]. Past research also documented higher risks of psychological distress associated with substance use, unhealthy diet, and physical inactivity among students [14, 20]. Thus, a considerable pool of literature suggests that psychological distress is related to unhealthy behaviors and practices.

Furthermore, SEP indicators may indirectly affect individuals’ mental health through lifestyle behaviors [21]. Although individuals are free to make certain lifestyle choices, such choices might be constrained by socio-economic and structural conditions. Available evidence suggests that health risk behaviors are socially patterned in that they are more prevalent and tend to co-occur in lower socio-economic groups [22]. For instance, individuals with lower education and income are found to adopt unhealthy behaviors, including smoking and alcohol consumption [23], which may negatively affect their motivation for enhancing mental well-being [24], whereas individuals with higher SEP have more awareness about their health which may facilitate their participation in health promotion activities such as physical activity or exercise, decreased substance use, and healthy diet [23]. Thus, individuals’ SEP may influence health risk and protective behaviors which in turn may affect their mental health [25, 26].

**Fig. 1:**
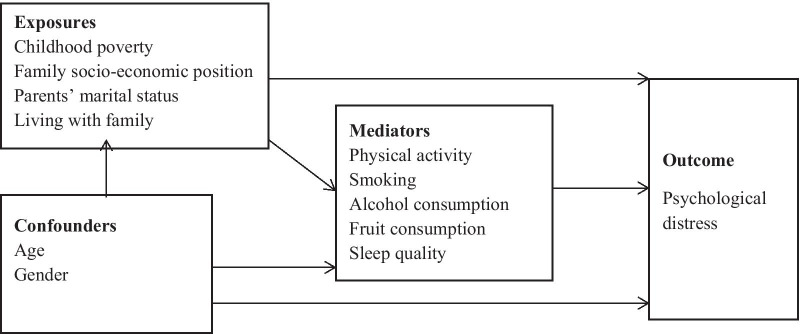
Conceptual framework of the study

While there is no dearth of literature that examined the social and behavioral predictors of psychological health, the mechanisms that may underlie the reported associations have not been adequately explored. In Bangladesh, only a few studies have investigated the association of psychological distress with social and lifestyle indicators [7, 27–29], which generally suffer from limited generalizability or insufficient statistical power. To our knowledge, no study has yet been undertaken in the context of Bangladesh to explore the mediating mechanisms pertaining to the social origins of psychological distress. From a policy perspective, a scientific understanding of the causal mechanisms is crucial to appropriately identify the entry points for interventions. Examining the mediating role of the well-established lifestyle factors affecting psychological distress (e.g., smoking, physical activity, alcohol drinking, diet, and quality sleep) is especially important as these risk factors are modifiable and easily amenable to interventions. Using a well-defined student sample drawn from multiple educational institutions in Bangladesh, the current study aimed to fill up the knowledge gap by systematically examining the associations of psychological distress with a broad set of social and lifestyle determinants, with a particular emphasis on the mediating role of the lifestyle factors behind the associations (See also Fig. 1).

## Methods

### Study sample

The study sample consists of the students, both males and females, who were pursuing their Bachelor or Master degrees at the universities/colleges located in Dhaka, the capital city of Bangladesh. Tertiary level education in Bangladesh is broadly aligned in three major streams: public university, private university, and national university. We used a multistage cluster sampling technique to recruit a representative sample from all three types of institutions. At first, we selected six educational institutions—two public universities, two private universities, and two colleges affiliated with the National University—from a comprehensive list of the Dhaka-based tertiary level educational institutions. Next, we randomly selected two departments from each of the six educational institutions, resulting in a total of 12 departments. Using social media platforms, we sent out a semi-structured questionnaire to the students from the selected departments during January–February 2020. The questionnaire was originally designed in English, but a translated Bengali version was administered for data collection. A total of 840 participants filled out the online questionnaire of which 88% (n = 742) had complete data on all the study variables. The participants provided informed consent before filling out the questionnaire. The study was approved by the Research Ethics Committee of the Department of Educational and Counselling Psychology at the University of Dhaka. All methodological procedures were accomplished in accordance with the relevant ethical guidelines and regulations.

### Measures

#### Outcome

The primary outcome variable in this study was psychological distress which we assessed by the 12-item General Health Questionnaire (GHQ-12) [30]. The GHQ-12 is a widely employed and well-validated screening instrument [31, 32] to measure common mental disorders (e.g., depression, anxiety, and stress) that disrupt the performance of an individual’s daily activities [33–35]. The GHQ-12 includes both positively and negatively worded items, e.g., “In the past two weeks, have you been able to enjoy your normal day to day activities?”; “Have you been feeling unhappy or depressed in the past two weeks?” Each item was graded on a 4-point Likert scale such as “much more than usual”, “more than usual”, “not more than usual”, and “not at all”. Although several procedures to sum up the GHQ-12 scores are available [32, 36], we employed the 0–0–1–1 scoring method which yield a total of 12 scores ranging between 0 and 12, with a higher score indicating greater psychological distress [37]. In the current study, the index showed a very good internal consistency with a Cronbach's alpha of 0.84. We used the mean GHQ-12 score, which was 3.9 in this study, as a threshold to determine psychological distress, as suggested by Goldberg [36].

#### Exposures

The exposures considered in the study were a range of social disadvantages originating from the participants’ family of origin: childhood poverty, family SEP, parents’ marital status, and living away from the family. To measure childhood poverty**,** participants were asked whether they experienced any serious financial difficulty in the family before they were 16 years old. The response options were coded as yes, no, and don’t remember. Family SEP was measured by asking the participants to assess their parents’ socio-economic status on a 5-point hierarchical scale and the responses were coded into 3 socio-economic groups: high (high/higher middle class), medium (intermediate middle class), and low (low/lower middle class). Parents’ marital status was categorized as married and unmarried (divorced/separated/widowed). Participants’ living status was measured by asking them to indicate their living arrangement at the time of the survey. Those who reported to be living with friends in student hostels or rented apartments were categorized as living away from the family of origin.

#### Mediators

The potential mediating variables included in the study were physical activity, smoking, alcohol consumption, fruit consumption, and sleep quality. To measure physical activity, participants were asked about the frequency of engagement in intensive physical activities (such as walking briskly, running, bicycling, swimming, playing sports, or any other activity) for at least 30 min at a time, that cause some increase in breathing or heart rate. Physical activity was coded into three categories: active (more than 3 times per week), moderately active (1–3 times per week), and inactive (never or less than 3 times per month). Smoking status was ascertained by two questions on current and former smoking, respectively. Participants who were reportedly not smoking cigarettes during the survey were further asked if they had smoked in the past for at least six months. The two variables were collapsed into a single variable which was categorized as: never smoker, current smoker, and former smoker. Alcohol consumption was assessed by asking whether participants drank alcohol (e.g., wine, beer, whisky, etc.) in the last one month and the responses were coded into three levels: never, sometimes (1–3 times per month), and regularly (more than once a week). As for fruit consumption, participants were asked how often they had fruits (such as bananas, guavas, mangos, pineapples, apples, oranges, jackfruits) in the past one month. The response items were coded as follows: rarely (never or fewer than 4 times per month), sometimes (1–3 times per week), and regularly (more than 3 times per week). The importance of fruit consumption as a core component of healthy diet was recognized by the World Health Organization [38] and it was frequently used as a lifestyle variable in the previous studies [39, 40]. The measure of poor sleep quality was assessed by a series of three questions related to: (a) difficulty in falling asleep for 30 min or longer, (b) frequent awakenings at night, and (c) early awakening in the morning and then having difficulty in going back to sleep. Each of the three questions had five answers: very often (1), often (2), sometimes (3), rarely (4), and never (5) which were collapsed into yes (often/very often) and no (never/rarely/sometimes) categories.

#### Control variables

The main variables considered as confounders in the analysis were age (continuous) and gender (male and female). Moreover, when modelling an association of an exposure or mediator with the outcome, relevant confounders were selected from the remaining set of exposures and/or mediators.

### Statistical analysis

Stata version 15.0 was used for data management and analysis. A description of the sample characteristics and the distribution of psychological distress by the study variables are presented as counts and proportions. The associations of the social and lifestyle characteristics with psychological distress were examined using the so-called modified Poisson regression analysis. The regression estimates were presented as risk ratios (RR) with 95% confidence intervals (CI). We used the robust sandwich estimators of variance to correct the standard errors of the Poisson regression models which are known to produce wider CIs. Poisson regression as a viable alternative to logistic regression was previously demonstrated in the context of a binary outcome of common prevalence i.e., > 10% [41, 42]. Because psychological distress was highly prevalent in our data (47%), we preferred the RRs over the odds ratios (OR) which would have inflated the relative risks. As there was no evidence of effect modification by gender, all analyses were carried out in the combined sample of males and females.

The statistical analyses were carried out at three stages. At first, we explored the associations between the social and the lifestyle characteristics using Pearson’s chi-square test. Next, we examined the associations of the lifestyle factors with psychological distress, both in minimally and multiply adjusted models. The minimally adjusted models controlled for age and gender while the multiply adjusted models additionally controlled for the social characteristics and mutually controlled for the lifestyle predictors. Finally, we investigated the associations between the social characteristics and psychological distress and further assessed to what degree these associations were mediated by the lifestyle factors. We drew on the commonly used change-in-estimate approach to assess the magnitude of mediation. Within this approach, we first estimated the confounder-adjusted total effect of the exposure. The direct effect was then obtained by statistically controlling for the mediator/s of interest. Any percentage change in the direct effect in comparison with the total effect was interpreted as the proportion mediated.

## Results

The mean age of the 742 students was 22.4 (SD = 2.3, range 18–30 years), with slightly more than half of the students being female (53.1%). The prevalence of psychological distress was 47%. Table 1 shows that the prevalence of psychological distress was higher in students who were males (54%) and experienced poverty during childhood (54%). Furthermore, the prevalence of psychological distress was relatively high among students who were physically inactive (52%), former smokers (62%), had irregular consumption of fruits (52%), and poor sleep quality (57%).

**Table 1:** Descriptive statistics of individual variables and by psychological distress

Characteristics	Total	Psychological distress		P-value*
		Yes	No	
	M (SD)/% (n)	M (SD)/% (n)	M (SD)/% (n)	
Age (Mean; Std. deviation)	22.4 (2.3)	22.2 (2.3)	22.7 (2.3)	0.007
Gender				
Male	46.9 (348)	54.3 (214)	45.7 (180)	0.001
Female	53.1 (394)	37.9 (132)	62.1 (216)	
Childhood poverty				
No	55.3 (410)	42.0 (172)	58.0 (238)	0.012
Yes	27.5 (204)	54.4 (111)	45.6 (93)	
Don’t remember	17.2 (128)	49.2 (63)	50.8 (65)	
Family socio-economic position				
High	30.2 (224)	46.0 (103)	54.0 (121)	0.185
Medium	59.0 (438)	45.2 (198)	54.8 (240)	
Low	10.8 (80)	56.3 (45)	43.8 (35)	
Parents’ marital status				
Married	92.6 (687)	47.3 (325)	52.7 (362)	0.192
Unmarried	7.4 (55)	38.2 (21)	61.8 (34)	
Living with family				
Yes	80.5 (597)	45.4 (271)	54.6 (326)	0.170
No	19.5 (145)	51.7 (75)	48.3 (70)	
Physical inactivity				
Inactive	41.8 (310)	52.3 (162)	47.7 (148)	0.014
Moderately active	24.9 (185)	38.9 (72)	61.1 (113)	
Active	33.3 (247)	45.3 (112)	54.7 (135)	
Smoking				
Never smoker	70.6 (524)	47.3 (248)	52.7 (276)	0.019
Current smoker	22.6 (168)	39.9 (67)	60.1 (101)	
Former smoker	6.7 (50)	62.0 (31)	38.0 (19)	
Alcohol consumption				
Never	89.9 (667)	47.4 (316)	52.6 (351)	0.359
Sometimes	8.5 (63)	38.1 (24)	61.9 (39)	
Regularly	1.6 (12)	50.0 (6)	50.0 (6)	
Fruit consumption				
Rarely	24.1 (179)	52.0 (93)	48.0 (86)	0.015
Sometimes	37.7 (280)	50.0 (140)	50.0 (140)	
Regularly	38.1 (283)	39.9 (113)	60.1 (170)	
Poor sleep quality				
No	63.3 (470)	40.9 (192)	59.1 (278)	0.001
Yes	36.7 (272)	56.6 (154)	43.4 (118)	
Total	100 (742)	46.6 (346)	53.4 (396)	

*Chi-square test for categorical variables and t-test for the continuous age variable

Table 2 shows the bivariate associations between the family social conditions and lifestyle factors. The chi-square tests suggest that students with a history of childhood poverty, compared to those who did not experience poverty in childhood, had a significantly higher prevalence of physical inactivity (p = 0.021), alcohol drinking (p = 0.012), and infrequent fruit consumption (p = 0.016). We also found that the students living with the family compared to those who live away were more likely to consume fruits regularly (42% versus 24%, p < 0.001). Moreover, the unhealthy lifestyles were overall more prevalent among the students with poorer family SEP and unmarried parents, but these differences were not statistically significant.

**Table 2:** Distribution of the lifestyle factors by family social conditions (n = 742)^#^

Lifestyle factors	Childhood poverty (%)			Family socio-economic position (%)			Parents’ marital status (%)		Living with family (%)	
	No	Yes	Don’t remember	High	Medium	Low	Married	Unmarried	Yes	No
Physical inactivity										
Inactive	44.6	37.3	39.8	38.8	43.6	40.0	41.5	45.5	42.4	39.3
Moderately active	22.4	24.0	34.4	28.1	23.3	25.0	25.0	23.6	24.0	29.0
Active	32.9	38.7	25.8	33.0	33.1	35.0	33.5	30.9	33.7	31.7
P for difference*	0.021			0.673			0.847		0.456	
Smoking										
Never smoker	72.4	69.6	66.4	77.7	67.6	67.5	69.7	81.8	71.7	66.2
Current smoker	22.0	23.0	24.2	17.4	25.1	23.8	23.1	16.4	21.6	26.9
Former smoker	5.6	7.4	9.4	4.9	7.3	8.8	7.1	1.8	6.7	6.9
P for difference	0.559			0.092			0.121		0.378	
Alcohol consumption										
Never	92.7	87.8	84.4	89.7	91.1	83.8	89.7	92.7	90.6	86.9
Sometimes/Regularly	7.3	12.3	15.6	10.3	8.9	16.3	10.3	7.3	9.4	13.1
P for difference*	0.012			0.134			0.469		0.182	
Fruit consumption										
Rarely	19.8	31.4	26.6	20.5	24.0	35.0	23.4	32.7	21.6	34.5
Sometimes	38.3	37.3	36.7	37.5	38.4	35.0	37.7	38.2	36.9	41.4
Regularly	42.0	31.4	36.7	42.0	37.7	30.0	38.9	29.1	41.5	24.1
P for difference*	0.016			0.107			0.212		0.001	
Poor sleep quality										
No	65.4	58.3	64.8	63.0	62.3	70.0	63.8	58.1	62.1	68.3
Yes	34.6	41.7	35.2	37.1	37.7	30.0	36.2	41.8	37.9	31.7
P for difference*	0.217			0.420			0.409		0.169	

^#^Column percentages are presented. *P-value obtained by Chi-square test

The results from the multivariable Poisson regression analyses on the association of lifestyle factors with psychological distress are provided in Table 3. The age- and gender-adjusted results in Model 1 showed an increased risk of psychological distress associated with former smoking status (RR: 1.44; 95% CI: 1.14, 1.81), rare fruit consumption (RR: 1.31; 95% CI: 1.07, 1.60), and poor sleep quality (RR: 1.35; 95% CI: 1.17, 1.57). Moderate level of physical activity was found to be protective of psychological distress (RR: 0.77; 95% CI: 0.62, 0.94). The statistical adjustments for family social conditions in Model 2 slightly attenuated the RRs for fruit consumption, former smoking, and moderate physical activity. When the lifestyle factors were further adjusted for each other in Model 3, the strengths of these associations remained largely unaffected, except for the association with fruit consumption that was no longer significant.

**Table 3:** Risk ratios of the associations between lifestyle factors and psychological distress (n = 742)

Lifestyle factors	Model 1	Model 2	Model 3
	RR (95% CI)	RR (95% CI)	RR (95% CI)
Physical inactivity			
Inactive (ref.)	1.00	1.00	1.00
Moderately active	0.77 (0.62, 0.94)	0.75 (0.61, 0.92)	0.76 (0.62, 0.94)
Active	0.91 (0.77, 1.08)	0.92 (0.78, 1.10)	0.96 (0.81, 1.14)
Smoking			
Never smoker (ref.)	1.00	1.00	1.00
Current smoker	1.09 (0.86, 1.39)	1.12 (0.89, 1.42)	1.13 (0.89, 1.44)
Former smoker	1.44 (1.14, 1.81)	1.39 (1.11, 1.75)	1.38 (1.09, 1.76)
Fruit consumption			
Rarely	1.31 (1.07, 1.60)	1.24 (1.02, 1.52)	1.18 (0.99, 1.41)
Sometimes	1.25 (1.04, 1.50)	1.23 (1.02, 1.47)	1.18 (0.96, 1.44)
Regularly (ref.)	1.00	1.00	1.00
Alcohol consumption			
Never (ref.)	1.00	1.00	1.00
Sometimes/Regularly*	0.99 (0.73, 1.33)	0.95 (0.70, 1.28)	0.90 (0.65, 1.23)
Poor sleep quality			
No (ref.)	1.00	1.00	1.00
Yes	1.36 (1.17, 1.58)	1.37 (1.19, 1.59)	1.35 (1.17, 1.57)

Model 1: Adjusted for age and gender

Model 2: Model 1 + adjustment for all family social variables

Model 3: Model 2 + mutual adjustment for all lifestyle factors

RR, Risk Ratio; CI, Confidence Interval

*Collapsed due to low number of cases

Table 4 presents the associations between the family social conditions and psychological distress as well as the mediating role of the lifestyle factors in the associations. It was found that students reporting childhood poverty had 1.31 times greater risk of psychological distress compared to students with no history of childhood poverty (95% CI: 1.11, 1.55). When physical inactivity, smoking, fruit consumption, alcohol consumption, and poor sleep quality were jointly accounted for, the estimated total effect of childhood poverty was reduced to 1.26 (95% CI: 1.06, 1.49), which is equivalent to 16% mediation. Compared to high family SEP, low family SEP showed a 1.36-fold higher risk of psychological distress, in which the lifestyle factors together mediated 8% (RR for Direct Effect: 1.33; 95% CI: 1.05, 1.68) of the total effect. Students who reported to be living away from the family had 28% greater risk of psychological distress compared to their counterparts living with the family (RR for Total Effect: 1.28, 95% CI: 1.07, 1.54). Statistical adjustments for the whole set of lifestyle factors did not mediate this association (RR for Direct Effect: 1.28, 95% CI: 1.06, 1.53). As far as the individual contributions of the lifestyle factors are concerned, fruit consumption appeared to be the strongest mediator which alone accounted for 10%, 11% and 18% of the associations of psychological distress with childhood poverty, low family SEP, and living away from family, respectively.

**Table 4:** Risk ratios of the associations between family social conditions and psychological distress: the mediating role of lifestyle factors (n = 742)

Family social conditions	Model 1: Age and gender-adjusted	Model 1 + Physical inactivity	Model 1 + Smoking	Model 1 + Fruit consumption	Model 1 + alcohol consumption	Model 1 + Poor sleep quality	Fully adjusted model: Model 1 + physical inactivity, smoking, fruit consumption, alcohol consumption, and sleep quality	
	RR (95% CI)	RR (95% CI)	RR (95% CI)	RR (95% CI)	RR (95% CI)	RR (95% CI)	RR (95% CI)	Change in RR (%) *
Childhood poverty								
No (ref.)	1.00	1.00	1.00	1.00	1.00	1.00	1.00	
Yes	1.31 (1.11, 1.55)	1.32 (1.12, 1.56)	1.30 (1.10, 1.54)	1.28 (1.08, 1.51)	1.31 (1.11, 1.55)	1.28 (1.08, 1.51)	1.26 (1.06, 1.49)	16
Don’t remember	1.21 (0.99, 1.49)	1.24 (1.01, 1.52)	1.20 (0.98, 1.47)	1.20 (0.98, 1.47)	1.22 (0.99, 1.49)	1.21 (0.99, 1.48)	1.19 (0.98, 1.46)	10
Family SEP								
High (ref.)	1.00	1.00	1.00	1.00	1.00	1.00	1.00	
Medium	1.04 (0.88, 1.24)	1.03 (0.86, 1.22)	1.03 (0.87, 1.23)	1.03 (0.87, 1.23)	1.04 (0.87, 1.24)	1.04 (0.87, 1.22)	1.02 (0.86, 1.20)	50
Low	1.36 (1.07, 1.73)	1.35 (1.06, 1.71)	1.34 (1.05, 1.71)	1.32 (1.04, 1.69)	1.36 (1.07, 1.73)	1.38 (1.09, 1.74)	1.33 (1.05, 1.68)	8
Parents’ marital status								
Married (ref.)	1.00	1.00	1.00	1.00	1.00	1.00	1.00	
Unmarried	0.77 (0.55, 1.08)	0.77 (0.55, 1.07)	0.78 (0.56, 1.10)	0.75 (0.53, 1.05)	0.77 (0.55, 1.08)	0.75 (0.54, 1.05)	0.75 (0.54, 1.05)	9
Living with family								
Yes (ref.)	1.00	1.00	1.00	1.00	1.00	1.00	1.00	
No	1.28 (1.07, 1.54)	1.29 (1.08, 1.54)	1.30 (1.09, 1.56)	1.23 (1.03, 1.48)	1.28 (1.07, 1.54)	1.30 (1.09, 1.55)	1.28 (1.06, 1.53)	0

RR, Risk Ratio; CI, Confidence Interval; SEP, Socio-economic Position

*Calculated using the formula: (Total Effect from model 1 − Direct Effect from fully adjusted model)/(Total Effect from model 1 − 1) * 100.

## Discussion

The current study aimed to investigate the association of psychological distress with family social circumstances and lifestyle factors among students studying in tertiary institutions in Bangladesh, and the possible mediating role of the lifestyle factors in the associations between social disadvantages and the risk of psychological distress. The estimated prevalence of psychological distress in the study was as high as 47%. The findings of multivariable Poisson regression analyses suggested that students with former smoking status and poor sleep quality had elevated risks of psychological distress. Regular fruit consumption and physical activity at its moderate level were found to be protective of psychological distress. Moreover, childhood poverty, low family SEP, and living away from the family were significantly associated with psychological distress and the estimated associations were not convincingly mediated by the lifestyle factors.

The estimated prevalence rate of psychological distress (47%) in the current study is fairly comparable with the rates previously reported by some studies conducted among students in Bangladesh (49%) [43] India (42%) [44], Ireland (42%) [20], and Syria (53%) [45]. There is, however, a large discrepancy in prevalence rates of psychological distress in students across studies and geographic regions. While the reported rates of psychological distress among students in Australia [46], Canada [47], Tanzania [48], and the United States (US) [49] were between 13 and 30%, some studies in Bangladesh [7], Ethiopia [8], and Norway [6] found much higher rates of psychological distress among students, ranging from 41 to 73%. A number of factors including different measurement tools and definitions of the construct, sampling variations, heterogeneity of age range, differential risk and protective elements, and socio-cultural dissimilarities may explain the variations in students’ psychological distress worldwide.

The observed associations between lifestyle factors and psychological distress in our study are broadly consistent with previous literature documenting worse psychological health among students with former smoking status [50], poor sleep quality [16, 17], fruit consumption [18], and a lack of physical activity [51]. The current smokers, however, did not show any higher risk of psychological distress compared to never smokers. The lack of an association among current smokers in our study can be due to simultaneous assessments of both smoking and psychological distress while a true causal association would naturally involve an induction period between the initiation of smoking and the manifestation of psychological distress that can be captured by a longitudinal study design. A Norwegian prospective investigation, for example, demonstrated that cigarette smoking at baseline was strongly associated with the onset of depression 11 years later [52].

The reported associations between family social circumstances and psychological distress are compatible with the international literature suggesting that students who had childhood poverty [53], low SEP [11, 27], and lived in hostels or rented houses [54] experienced higher psychological distress. Mental distress, which arises from childhood poverty, may continue to increase as children grow up [55], which could subsequently influence SEP indicators, including their academic performance and living in hostels or rented houses [54]. The students migrating from rural areas to Dhaka city generally leave their families behind and live in hostels and rented houses with suboptimal living conditions. Typically, they are socio-economically disadvantaged compared to their counterparts who live with the family in the city, and are faced with unique challenges including financial constraints, adaptation to urban lifestyle, and loneliness [56], which may negatively impact upon their psychological wellbeing. Living away from the family may also mean less control of the family over the lifestyle of the students. Moreover, the students not living with their family may have limited opportunity to eat healthy diet [57]. This has been partly evident in our study which demonstrates that regular fruit consumption is more common among students living with their family and that nearly one-fifth of the observed association was explained by fruit consumption.

While the lifestyle factors such as former smoking status, poor sleep quality, physical activity and fruit consumption were strongly associated with psychological distress in the current study, the same factors altogether explained, only to small extents, the social differences in psychological distress. We did not come across any single study in Bangladesh to compare this finding with. However, international studies have yielded inconsistent evidence when it comes to quantifying the magnitude of mediation of the lifestyle mechanisms underlying the SEP-mental health associations [25, 58, 59]. For example, Groffen and colleagues [58] showed that lifestyle factors (smoking, alcohol consumption, body mass index, and physical activity) explained less than 10% of the association between depression and SEP—a finding which is largely in agreement with the finding of the current study. Bøe and colleagues [59] found that people’s sleep problems alone mediated one-third of the association between low family SEP and poor mental health. Since the contribution of the lifestyle factors to the associations between family social circumstances and psychological distress was found to be minimal in our study, we believe that there might be other possible pathways through which social circumstances affect students’ psychological distress.

There are at least three potential pathways through which socio-economic circumstances may affect psychological distress. First, people with higher SEP may have better cognitive ability and are generally more conscious of their mental health, which may enable them to participate in health promotion actions [23]. Second, individuals with low SEP have more potential to experience stressful life situations which may affect their mental health [60]. Third, the individuals with better social position usually have greater access to material and social resources [61] which can influence their health behaviors and practices that directly affect psychological health [21]. This suggests that the relationships between psychological distress and social and lifestyle factors are complex, warranting further investigations that should encompass a wide range of mediators.

## Strengths and limitations

Our study adds to the limited international literature examining the mediating mechanisms of the parental SEP-mental health association in early adulthood and provides novel evidence in the context of the Bangladesh society. The multi-institute sample is an advantage offering greater generalizability to the study findings when compared to previous studies in Bangladesh based on smaller samples from single educational institutions [29, 43, 62–64]. Furthermore, some of the predictors of psychological distress (such as fruit consumption, alcohol consumption, childhood poverty, and living away from the family) among tertiary students in the present study have been reported for the first time in Bangladesh.

However, the findings of the study should be interpreted in the context of the following limitations: First, due to cross-sectional research design, we could not rule out the possibility of reverse causality. Although it is plausible to assume that the social exposures we studied temporally precede the lifestyle factors, the relationships between psychological distress and lifestyle factors are most likely to be bidirectional. For example, while regular physical activity can improve mental well-being [25], sick individuals are more likely to be physically inactive. Second, psychological distress, social circumstances, and lifestyle behaviors were all self-reported, which could result in misclassification of the exposures, mediators, and the outcome. Moreover, the retrospective assessment of childhood poverty may lead to recall bias in the analysis provided that the students experiencing poverty in childhood reported it less accurately than their richer counterparts, although we tried to circumvent this issue by introducing a “don’t remember” category in the response options. Third, our mediation analysis is based on traditional regression modelling which is criticised for producing biased mediation parameters in the presence of exposure-mediator and mediator-mediator interactions [65, 66]. We did not detect any interaction between the social and lifestyle factors regarding psychological distress and hence the potential bias originating from interactions is expected to be minimal. However, future research should employ prospective design and counterfactual mediation framework to provide more valid insights into the causal mechanisms underlying the social inequalities in psychological health.

## Conclusions

We found increased risks of psychological distress in students with disadvantaged social backgrounds and unhealthy lifestyles, such as childhood poverty, low SEP, not living with the family, former smoking status, poor sleep quality, and irregular fruit consumption. The observed associations between social disadvantages in the family and psychological distress were weakly mediated by the lifestyle factors. Thus, while interventions aiming to promote healthy lifestyles may be useful to minimize the burden of psychological distress on an absolute level, our findings highlight the need for ameliorating the social and living conditions at the start of life to reduce the rich-poor gaps in psychological distress.

## Data Availability

The authors are not allowed to share the data publicly due to ethical reasons. However, the de-identified data used in the current study is accessible from the corresponding author upon reasonable requests.

## References

[1] Atkinson SR (2020). Elevated psychological distress in undergraduate and graduate entry students entering first year medical school. PLoS ONE.

[2] Jarrad T, Dry M, Semmler C, Turnbull D, Chur-Hansen A (2019). The psychological distress and physical health of Australian psychology honours students. Aust Psychol.

[3] Pratt LA (2009). Serious psychological distress, as measured by the K6, and mortality. Ann Epidemiol.

[4] Chiu M, Lebenbaum M, Cheng J, de Oliveira C, Kurdyak P (2017). The direct healthcare costs associated with psychological distress and major depression: A population-based cohort study in Ontario, Canada. PLoS ONE.

[5] Oksanen A, Laimi K, Björklund K, Löyttyniemi E, Kunttu K (2017). A 12-year trend of psychological distress: national study of Finnish university students. Cent Eur J Publ Health.

[6] Knapstad M, Sivertsen B, Knudsen AK, Smith ORF, Aarø LE, Lønning KJ, et al. Trends in self-reported psychological distress among college and university students from 2010 to 2018. Psychol Med. 2019:1–9 10.1017/S0033291719003350 PMC7958482 31779729

[7] Mamun MA, Akter S, Hossain I, Faisal MTH, Rahman MA, Arefin A, et al. Financial threat, hardship and distress predict depression, anxiety and stress among the unemployed youths: A Bangladeshi multi-cities study. J Affect Disord. 2020. 10.1016/j.jad.2020.06.075 32791351

[8] Dachew BA, Bisetegn TA, Gebremariam RB (2015). Prevalence of mental distress and associated factors among undergraduate students of University of Gondar, Northwest Ethiopia: a cross-sectional institutional based study. PLoS ONE.

[9] Imran N, Tariq KF, Pervez MI, Jawaid M, Haider II (2016). Medical students’ stress, psychological morbidity, and coping strategies: a cross-sectional study from Pakistan. Acad Psychiatry.

[10] Hope V, Henderson M (2014). Medical student depression, anxiety and distress outside North America: a systematic review. Med Educ.

[11] Divaris K, Mafla AC, Villa-Torres L, Sanchez-Molina M, Gallego-Gomez CL, Velez-Jaramillo LF (2013). Psychological distress and its correlates among dental students: a survey of 17 Colombian dental schools. BMC Med Educ.

[12] Hakami RM (2018). Prevalence of psychological distress among undergraduate students at Jazan University: a cross-sectional study. Saudi J Med Med Sci.

[13] Sokratous S, Merkouris A, Middleton N, Karanikola M (2014). The prevalence and socio-demographic correlates of depressive symptoms among Cypriot university students: a cross-sectional descriptive co-relational study. BMC Psychatry.

[14] Bore M, Kelly B, Nair B (2016). Potential predictors of psychological distress and well-being in medical students: a cross-sectional pilot study. Adv Med Educ Pract.

[15] Man X, Cao H. Prevalence and protective factors of psychological distress among left-behind children in rural China: a study based on national data. J Child Fam Stud. 2020:1–10.

[16] Milojevich HM, Lukowski AF (2016). Sleep and mental health in undergraduate students with generally healthy sleep habits. PLoS ONE.

[17] Rezaei M, Khormali M, Akbarpour S, Sadeghniiat-Hagighi K, Shamsipour M (2018). Sleep quality and its association with psychological distress and sleep hygiene: a cross-sectional study among pre-clinical medical students. Sleep Sci.

[18] Pengpid S, Peltzer K. Psychological distress and its associated factors among school-going adolescents in Tanzania. Psychol Stud. 2020:1–8.

[19] Balogun O, Koyanagi A, Stickley A, Gilmour S, Shibuya K (2014). Alcohol consumption and psychological distress in adolescents: a multi-country study. J Adolesc Health.

[20] Deasy C, Coughlan B, Pironom J, Jourdan D, Mannix-McNamara P (2014). Psychological distress and coping amongst higher education students: a mixed method enquiry. PLoS ONE.

[21] Wang J, Geng L (2019). Effects of socioeconomic status on physical and psychological health: lifestyle as a mediator. Int J Environ Res Public Health.

[22] Falkstedt D, Möller J, Zeebari Z, Engström K (2016). Prevalence, co-occurrence, and clustering of health-risk behaviors among people with different socio-economic trajectories: a population-based study. Prev Med.

[23] Schüz B, Brick C, Wilding S, Conner M (2020). Socioeconomic status moderates the effects of health cognitions on health behaviors within participants: two multibehavior studies. Ann Behav Med.

[24] Jury M, Smeding A, Stephens NM, Nelson JE, Aelenei C, Darnon C (2017). The experience of low-SES students in higher education: Psychological barriers to success and interventions to reduce social-class inequality. J Soc Issues.

[25] Kim J (2011). The mediating effects of lifestyle factors on the relationship between socioeconomic status and self-rated health among middle-aged and older adults in Korea. Int J Aging Hum Dev.

[26] Pascoe M, Bailey AP, Craike M, Carter T, Patten R, Stepto N, et al. Physical activity and exercise in youth mental health promotion: a scoping review. BMJ Open Sport Exerc Med. 2020;6. 10.1136/bmjsem-2019-000677 PMC7010991 32095272

[27] Islam FMA (2019). Psychological distress and its association with socio-demographic factors in a rural district in Bangladesh: a cross-sectional study. PLoS ONE.

[28] Islam FMA, Walton A. Tobacco smoking and use of smokeless tobacco and their association with psychological distress and other factors in a rural district in Bangladesh: a cross-sectional study. J Environ Public Health. 2019;2019. 10.1155/2019/1424592 PMC6918939 31885635

[29] Islam MA, Hossin MZ (2016). Prevalence and risk factors of problematic internet use and the associated psychological distress among graduate students of Bangladesh. Asian J Gamb Issues Public Health.

[30] Goldberg D (1972). The detection of psychiatric illness by questionnaire.

[31] Goldberg DP, Gater R, Sartorius N, Ustun TB, Piccinelli M, Gureje O (1997). The validity of two versions of the GHQ in the WHO study of mental illness in general health care. Psychol Med.

[32] Anjara S, Bonetto C, Van Bortel T, Brayne C (2020). Using the GHQ-12 to screen for mental health problems among primary care patients: psychometrics and practical considerations. Int J Ment Health Syst.

[33] Endsley P, Weobong B, Nadkarni A (2017). The psychometric properties of GHQ for detecting common mental disorder among community dwelling men in Goa. India Asian J Psychiatr.

[34] Goldberg D. Manual of the general health questionnaire: Nfer Nelson; 1978.

[35] El-Metwally A, Javed S, Razzak HA, Aldossari KK, Aldiab A, Al-Ghamdi SH (2018). The factor structure of the general health questionnaire (GHQ12) in Saudi Arabia. BMC Health Serv Res.

[36] Goldberg D, Oldehinkel T, Ormel J (1998). Why GHQ threshold varies from one place to another. Psychol Med.

[37] McDowell I. Measuring health: a guide to rating scales and questionnaires: Oxford University Press, USA; 2006.

[38] WHO. Promoting fruit and vegetable consumption around the world: WHO; 2004. https://www.who.int/dietphysicalactivity/fruit/en/.

[39] Loef M, Walach H (2012). The combined effects of healthy lifestyle behaviors on all cause mortality: a systematic review and meta-analysis. Prev Med.

[40] Larsson SC, Kaluza J, Wolk A (2017). Combined impact of healthy lifestyle factors on lifespan: two prospective cohorts. J Intern Med.

[41] Hossin MZ, Östergren O, Fors S (2019). Is the association between late life morbidity and disability attenuated over time? Exploring the dynamic equilibrium of morbidity hypothesis. J Gerontol: Series B.

[42] Zou G (2004). A modified Poisson regression approach to prospective studies with binary data. Am J Epidemiol.

[43] Rafi M, Mamun MA, Hsan K, Hossain M, Gozal D (2019). Psychological implications of unemployment among Bangladesh civil service job seekers. Front Psychiatry.

[44] Sathyanath MS, Kundapur R (2020). Epidemiological correlates of psychological distress in a rural community of South India: a cross-sectional study. Indian J Community Med.

[45] Al Saadi T, Addeen SZ, Turk T, Abbas F, Alkhatib M (2017). Psychological distress among medical students in conflicts: a cross-sectional study from Syria. BMC Med Educ.

[46] Larcombe W, Finch S, Sore R, Murray CM, Kentish S, Mulder RA (2016). Prevalence and socio-demographic correlates of psychological distress among students at an Australian university. Stud High Educ.

[47] Meckamalil C, Brodie L, Hogg-Johnson S, Carroll LJ, Jacobs C, Côté P. The prevalence of anxiety, stress and depressive symptoms in undergraduate students at the Canadian Memorial Chiropractic College. J Am Coll Health. 2020:1–6. 10.1080/07448481.2020.1751173 32369713

[48] Mboya IB, John B, Kibopile ES, Mhando L, George J, Ngocho JS (2020). Factors associated with mental distress among undergraduate students in northern Tanzania. BMC Psychiatry.

[49] Becerra MB, Becerra BJ (2020). Psychological distress among college students: role of food insecurity and other social determinants of mental health. Int J Environ Res Pu.

[50] Coskun O, Ocalan AO, Ocbe CB, Semiz HO, Budakoglu I (2019). Depression and hopelessness in pre-clinical medical students. Clin Teach.

[51] Perales F, Pozo-Cruz Jd, Pozo-Cruz Bd. Impact of physical activity on psychological distress: a prospective analysis of an Australian national sample. Am J Public Health 2014;104:e91-e7. 10.2105/AJPH.2014.302169 PMC4232115 25322296

[52] Klungsøyr O, Nygård JF, Sørensen T, Sandanger I (2006). Cigarette smoking and incidence of first depressive episode: an 11-year, population-based follow-up study. Am J Epidemiol.

[53] Evans GW (2016). Childhood poverty and adult psychological well-being. PNAS.

[54] Kunwar D, Risal A, Koirala S (2016). Study of depression, anxiety and stress among the medical students in two medical colleges of Nepal. Kathmandu Univ Med J.

[55] Twenge JM, Cooper AB, Joiner TE, Duffy ME, Binau SG (2019). Age, period, and cohort trends in mood disorder indicators and suicide-related outcomes in a nationally representative dataset, 2005–2017. J Abnorm Psychol.

[56] Khan S, Shahriar MS, Jahan S, Zayed NM. The challenges of students from rural backgrounds in urban institutions for tertiary education: a case study on students’ migration to Dhaka City. Int J Manag. 2020;11.

[57] National Research Council. Supplemental nutrition assistance program: examining the evidence to define benefit adequacy: National Academies Press; 2013. 24901188

[58] Groffen DA, Koster A, Bosma H, Van Den Akker M, Kempen GI, Van Eijk JTM (2013). Unhealthy lifestyles do not mediate the relationship between socioeconomic status and incident depressive symptoms: the health ABC study. Am J Geriatr Psychiatry.

[59] Bøe T, Hysing M, Stormark KM, Lundervold AJ, Sivertsen B (2012). Sleep problems as a mediator of the association between parental education levels, perceived family economy and poor mental health in children. J Psychosom Res.

[60] Reiss F, Meyrose A-K, Otto C, Lampert T, Klasen F, Ravens-Sieberer U (2019). Socioeconomic status, stressful life situations and mental health problems in children and adolescents: results of the German BELLA cohort-study. PLoS ONE.

[61] Pearce A, Dundas R, Whitehead M, Taylor-Robinson D (2019). Pathways to inequalities in child health. Arch Dis Child.

[62] Mamun MA, Hossain MS, Moonajilin MS, Masud MT, Misti JM, Griffiths MD (2020). Does loneliness, self-esteem and psychological distress correlate with problematic internet use? A Bangladeshi survey study. Asia Pac Psychiatry.

[63] Al Mamun M, Griffiths MD (2019). The association between Facebook addiction and depression: a pilot survey study among Bangladeshi students. Psychiat Res.

[64] Sultana N (2011). Stress and depression among undergraduate medical students of Bangladesh. Bangl J Med Educ.

[65] Richiardi L, Bellocco R, Zugna D (2013). Mediation analysis in epidemiology: methods, interpretation and bias. Int J Epidemiol.

[66] Hossin MZ, Koupil I, Falkstedt D (2019). Early life socioeconomic position and mortality from cardiovascular diseases: an application of causal mediation analysis in the Stockholm Public Health Cohort. BMJ Open.

